# Mapping of Agricultural Subsurface Drainage Systems Using a Frequency-Domain Ground Penetrating Radar and Evaluating Its Performance Using a Single-Frequency Multi-Receiver Electromagnetic Induction Instrument †

**DOI:** 10.3390/s20143922

**Published:** 2020-07-14

**Authors:** Triven Koganti, Ellen Van De Vijver, Barry J. Allred, Mogens H. Greve, Jørgen Ringgaard, Bo V. Iversen

**Affiliations:** 1 Department of Agroecology, Aarhus University, Blichers Allé 20, 8830 Tjele, Denmark; bo.v.iversen@agro.au.dk (B.V.I.); mogensh.greve@agro.au.dk (M.H.G.); 2 Research Group Soil Spatial Inventory Techniques, Department of Environment, Ghent University, Coupure Links 653, 9000 Gent, Belgium; ervdevij.vandevijver@ugent.be; 3 USDA/ARS Soil Drainage Research Unit, 590 Woody Hayes Drive, Room 234, Columbus, OH 43210, USA; barry.allred@usda.gov; 4 Rambøll, Hannemanns Allé 53, 2300 Copenhagen, Denmark; jri@ramboll.dk

**Keywords:** frequency-domain, ground penetrating radar, electromagnetic induction, penetration depth, inversion, non-destructive techniques, agricultural drainage systems

## Abstract

Subsurface drainage systems are commonly used to remove surplus water from the soil profile of a poorly drained farmland. Traditional methods for drainage mapping involve the use of tile probes and trenching equipment that are time-consuming, labor-intensive, and invasive, thereby entailing an inherent risk of damaging the drainpipes. Effective and efficient methods are needed in order to map the buried drain lines: (1) to comprehend the processes of leaching and offsite release of nutrients and pesticides and (2) for the installation of a new set of drain lines between the old ones to enhance the soil water removal. Non-invasive geophysical soil sensors provide a potential alternative solution. Previous research has mainly showcased the use of time-domain ground penetrating radar, with variable success, depending on local soil and hydrological conditions and the central frequency of the specific equipment used. The objectives of this study were: (1) to test the use of a stepped-frequency continuous wave three-dimensional ground penetrating radar (3D-GPR) with a wide antenna array for subsurface drainage mapping and (2) to evaluate its performance with the use of a single-frequency multi-receiver electromagnetic induction (EMI) sensor in-combination. This sensor combination was evaluated on twelve different study sites with various soil types with textures ranging from sand to clay till. While the 3D-GPR showed a high success rate in finding the drainpipes at five sites (sandy, sandy loam, loamy sand, and organic topsoils), the results at the other seven sites were less successful due to the limited penetration depth of the 3D-GPR signal. The results suggest that the electrical conductivity estimates produced by the inversion of apparent electrical conductivity data measured by the EMI sensor could be a useful proxy for explaining the success achieved by the 3D-GPR in finding the drain lines.

## 1. Introduction

**Figure 1:**
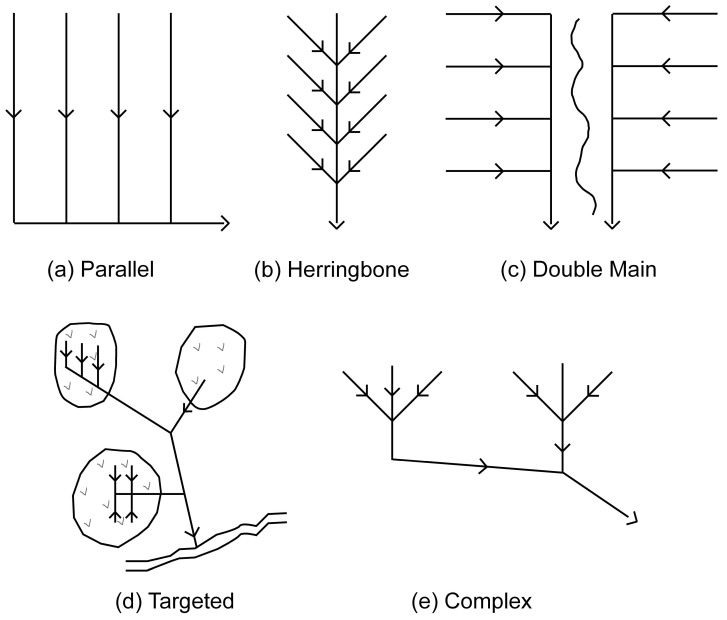
Commonly used subsurface drainage system patterns (modified from [18,19]).

The installation of subsurface drainage systems comprised of buried drainage pipe networks has been a common practice for decades in order to enhance the water removal capability of naturally poorly drained soils. Some of the most productive agricultural regions in the world are a result of subsurface drainage practices [1,2]. Subsurface drainage provides many agronomic, economic, and environmental benefits by lowering the water table enhancing plant productivity, and improving the trafficability and timeliness of field operations thereby increasing the crop yields [3,4]. However, the excessive leaching of nutrients and pesticides through the percolation of solutes through the root zone to drainage pipes is a potential risk for eutrophication and contamination of the surface water bodies [5,6,7]. In Denmark, considerable attention is being directed towards the role of drainage systems in the transport and leaching of nutrients and pesticides to the aquatic environment [8]. Nevertheless, due to limited information on subsurface drainage installations, it is difficult to understand the hydrology and solute dynamics and plan effective mitigation strategies, such as constructed wetlands, saturated buffer zones, bioreactors, and nitrate and phosphate filters [9,10,11,12,13]. Apart from these environmental aspects, there also are practical reasons motivating investment in improved drain line mapping. To enhance the drainage efficiency in agricultural areas with established drainage systems, new drain lines can be installed in between the old ones, which requires knowledge of the precise location of the latter [14,15]. Additionally, dysfunctional drain pipes need to be accurately located for the farmers to get them repaired [16,17]. Old drainage systems often stay in place, even if no longer used or dysfunctional, as it is neither economical nor practical to remove them [5]. Figure 1 shows the typical patterns followed for subsurface drainage installations, yet, the drainage documentation is often lacking or outdated. 

Traditional methods of drainage mapping involve the use of tile probes and/or trenching equipment. While tile probing is localized and discrete, making it time-consuming and extremely tedious to apply at large spatial scales, using trenching equipment is more spatially comprehensive, but also exceedingly invasive, which may lead to damaging the existing drainage network, requiring costly repairs [17]. Non-invasive geophysical soil sensors provide an effective and efficient alternative to these problems. Previous research mainly shows the use of time-domain impulse ground penetrating radar (GPR), with variable success depending on the local soil and hydrological conditions and their compatibility with the specific equipment used (e.g., chosen center frequency) [14,15,16]. Moreover, the detection rate depends on the antenna orientation relative to the drain line directional trend [20]. Therefore, it is necessary to perform the survey along multiple parallel transects [15] or following spiral and serpentine transects [17] in order to confirm the presence of a drain line and ascertain its orientation.

Recent technological advances in proximal sensing techniques have enabled the collection of high-resolution, exhaustive datasets in three-dimensional (3D) space. This includes the development of 3D multi-channel [21,22,23,24,25,26] and multi-frequency GPR systems [22,26], as well as multi-receiver [27,28,29] and multi-frequency [30,31,32] electromagnetic induction (EMI) sensors. When applied in combination, these techniques provide complementary information and therefore support a comprehensive analysis of the subsurface. Some of the examples include, but are not limited to, investigation of industrial and urban soil contamination [33,34], characterization of agricultural soil morphology [35,36], unexploded ordnance detection and discrimination [37,38,39], and reconstruction of archaeological landscapes [40,41].

In this study, we present the use of a stepped-frequency continuous wave (SFCW) 3D-GPR (GeoScope Mk IV 3D-Radar with DXG1820 antenna array) in combination with a multi-receiver EMI sensor (DUALEM) for subsurface drainage mapping. The 3D-GPR system with a wide antenna array offers more flexibility for application to different (sub)surface conditions and effective coverage of 3D space. The EMI sensor simultaneously provides information regarding the apparent electrical conductivity (EC_a_) for different soil volumes, corresponding to different depths. Both sensors were mounted in a motorized survey configuration with real-time geo-referencing. This sensor combination was evaluated on twelve different study sites with various soil types, ranging from sand to clay till. The main goals of this research were: 1) to investigate the suitability of 3D-GPR for drain line mapping on different soil types and 2) evaluate whether electrical conductivity (EC) estimates derived from the EC_a_ measurements of the EMI sensor can act as a suitable proxy to explain the success of 3D-GPR for this purpose. The latter was achieved by proposing a novel methodology for computing the 3D-GPR global and localized penetration depths (PDs) and subsequently assessing the quantitative relationship between the EC estimates and localized PDs. Even when applied at a coarser survey resolution, EMI surveys are recommended for initial exploration, as they are cost-effective and more robust to environmental conditions than GPR and they can provide highly relevant information on the spatial variability of soil texture [42,43]. In addition, EMI surveys have an established reputation for their widespread use in agricultural applications [44,45,46] and they have proven successful for drainpipe detection in saline (high EC) soils [47,48,49]. The rationale for this research is based on the notion that soil EC governs the attenuation of electromagnetic waves (i.e. high signal attenuation in high EC areas) and, hence, controls the PD of the GPR signal [50].

## 2. Materials and Methods

### 2.1. Study Sites

**Figure 2:**
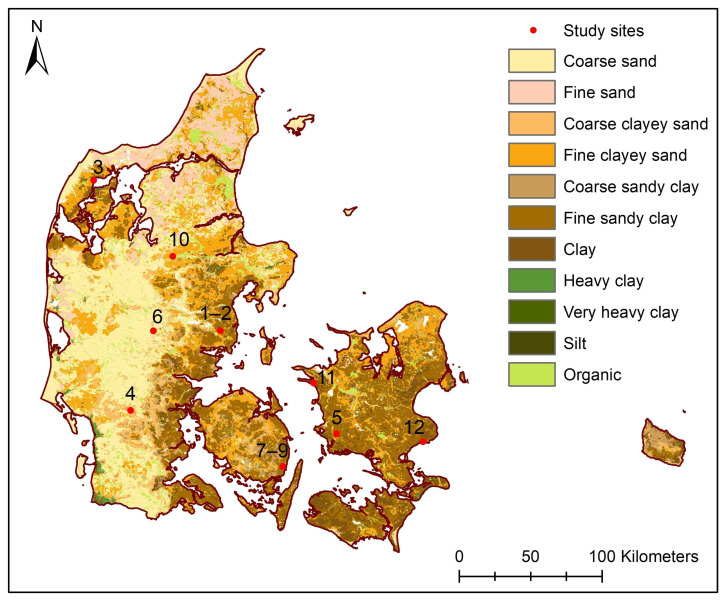
Map of Denmark showing the study sites’ location and soil types according to the Danish Soil Classification [58].

The selected study sites (Figure 2) are Fensholt—upland and lowland area (1–2), Silstrup (3), Estrup (4), Faardrup (5), Holtum (6), Lillebæk-1, 2, and 3 (7–9), Juelsgaard (10), Kalundborg (11), and Lund (12). The sites were selected to reflect variable soil textures and correspondingly variable soil water dynamics, and drainage system structures. Except for Holtum and Kalundborg, for each of these sites, digitized drainage maps were available, allowing to evaluate the performance of the 3D-GPR system.

Table 1 provides the summary of study site locations and soil types. The Fensholt catchment is characterized by a clayey and organic soil in the upland and lowland area, respectively, and it is heavily tile-drained for its agricultural land use [51]. Silstrup, Estrup, and Faardrup are sites monitored in the framework of the Danish Pesticide Leaching Assessment Programme (PLAP). The soil at each of these sites consists of sandy-loamy topsoil underlain by a clay till/sandy clay till subsoil. A more detailed description of the PLAP study sites can be found in [52]. Thick sedimentary deposits of sand and silt characterize the Holtum site [53]. The sites at Lillebæk, a clayey till watershed, are mostly characterized by a sand-mixed clayey soil [54]. The Juelsgaard site has loamy sand to sandy loam soil, with an intermediate layer of coarse sand and clay till in the subsoil. The soil at Kalundborg consists of sandy loam in the topsoil, an intermediate layer of organic material, and sandy clay till in the subsoil [55]. Lund is a new study site being monitored under the PLAP project and it consists of clayey sand topsoil and clay till in the subsoil [56]. Based on the World Reference Base for Soil Resources International soil classification system [57], the soils can be classified as a Phaeozem at Fensholt upland, Silstrup, Estrup, Faardrup, Lillebæk-2, and Kalundborg, Histosol at Fensholt lowland, Podzol at Holtum, Acrisol at Jueslgaard, and Luvisol at Lillebæk-1, 3, and Lund sites.

### 2.2. The 3D-GPR Instrument and Survey

A surface GPR system essentially consists of a transmitter and a receiver antenna. The transmitter antenna emits electromagnetic energy into the ground and the receiver antenna records the energy reflected and scattered back to the surface. The GPR wave propagation is mainly controlled by the two electric properties, relative dielectric permittivity (RDP) and EC, if the magnetic permeability can be assumed to be constant [60]. If the propagating wave encounters a subsurface contrast in RDP, energy is reflected back to the surface, the amount of which is proportional to the degree of this contrast. The RDP also governs the GPR wave velocity and, hence, the time between the transmission and detection of a reflection (two-way travel time) is proportional to the depth of the RDP contrast. The EC determines the GPR (Ohmic) signal attenuation and in lossy dispersive media, such as soils, the intrinsic attenuation increases with an increase in the frequency of the GPR waves [61,62,63]. The PD of a GPR system can be defined as the depth at which the signal reaches the background radio frequency (RF) noise floor [64] and it is mainly controlled by soil EC because signal attenuation is a major limiting factor. Other factors that control the signal-to-noise ratio (SNR) and the data quality include energy loss at the antennas, loss from reflections from contrasts in RDP, and loss due to absorption, scattering, and geometric spreading, which depends on the soil type and RDP [65]. Furthermore, the resolution of a GPR system is inversely proportional to its frequency bandwidth. Hence, the choice of bandwidth has to be made based on the resolution that is necessary for detecting the features of interest. While including higher frequencies generally improves the resolution, they also attenuate quickly and, therefore, have a shallow PD [26,61]. This limitation results in an important trade-off between the desired resolution and PD. In general, the resolution of the measurement signal decreases as it propagates through the subsurface due to the frequency-dependent attenuation of the GPR waves. Finally, the antennas orientation relative to the anomaly can have a significant effect on the detectability of the latter due to wave polarization, as the GPR waves are vectorial in nature [20,61].

**Figure 3:**
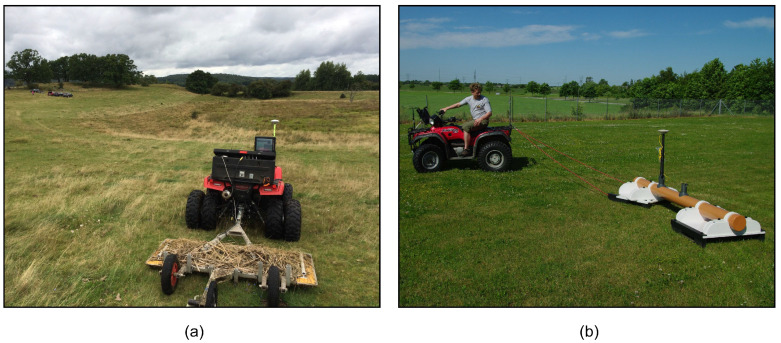
Survey configuration of: (**a**) the three-dimensional ground penetrating radar (3D-GPR) instrument and (**b**) the DUALEM-21S sensor.

The most common GPR systems are time-domain GPRs; however, we used a frequency-domain 3D-GPR system. The 3D-GPR (Figure 3a) that was used in this study was a stepped-frequency continuous wave (SFCW) GeoScope Mk IV 3D-Radar with DXG1820 antenna array (3d-Radar AS, Trondheim, Norway) covering a wide frequency interval of 60–3000 MHz. The antenna array is ground-coupled and it consists of 21 bow-tie monopole antennas arranged so that data are simultaneously recorded along 20 channels with a uniform spacing of 0.075 m, resulting in a total scan width of 1.5 m. The wide frequency coverage of the GeoScope Mk IV offers the flexibility to adjust the bandwidth depending on the desired resolution and the depth of interest under different (sub)surface conditions. In addition, the wide-area swathe of the antenna array and its ground-coupled configuration enables effective coverage of 3D space and ensures maximum energy coupling with the subsurface [66]. The main difference between time-domain and frequency-domain GPR systems lies in the way that the transmission signal is generated. In a time-domain GPR system, a short wave pulse of a limited bandwidth (typically characterized by the system’s center frequency) is transmitted at regular intervals and the reflected energy is recorded as a function of time. Contrarily, in an SFCW system, a sinusoidal waveform is transmitted continuously and the frequency is modulated in linear increments. In this way, energy is focused on each frequency step in turn, over a certain dwell time, with the coherent recording of the reflected energy at the receiver in frequency-domain. This provides an overall improved SNR and optimal PD [67].

In our surveys, the 3D-GPR system was mounted on an all-terrain vehicle (ATV) and geo-referencing was done using a real-time kinematic (RTK) Global Navigation Satellite System (GNSS) with sub-decimeter accuracy. The RTK/GNSS setup was installed at the back of the ATV (Figure 3a), thus requiring a correction of the recorded positions. An odometer wheel attached behind the antenna array controlled the measurement interval along the survey lines and the measurements were made over the full frequency bandwidth with variable frequency step size, dwell time, and time window. The step size determines the number of discrete frequency steps covered; the dwell time regulates the amount of time that is spent on each frequency step and the time window controls the total time window within which the GPR return signal is recorded.

Table 1 shows the dates and three-days total rainfall prior to the 3D-GPR surveys performed at different sites. At all of the sites, the surveys were performed without consideration of the ground wetness level from prior rainfall, and the survey direction was chosen depending on the long axis of field, tillage direction, and/or the expected orientation of the drainpipes. As a general protocol, the data were recorded as multiple subsets of smaller regions spanning the survey area to limit the file size and prevent the files from being corrupted. The surveys were performed on multiple occasions at the sites Fensholt lowland and Estrup. At Fensholt lowland, this was done in order to assess the effect of different environmental conditions and survey configurations on the quality of GPR survey results [68]. At Estrup, this was done to evaluate different driving directions, i.e. along the tillage direction and parallel to the drain line orientation, and vice versa. Full data coverage was obtained when driving along the till direction, while only partial coverage was obtained across the till direction due to rough and uneven surface. Except for Holtum, the rest of the sites were visited only once during the late summer to autumn period depending on the site availability. At Holtum, the data were acquired in Jan. 2016 when the ground was frozen, as this area is generally wet due to a nearby stream, potentially causing problematic surface conditions to drive on with an ATV. Nevertheless, as there was only a thin layer of snow, this resulted in a rough and uneven surface, and the survey could only be done at a very slow speed, explaining the limited coverage of the site as compared to originally planned.

### 2.3. The 3D-GPR Data Processing, Global and Localized Penetration Depth

The processing of data was done using the 3D-Radar Examiner software v 3.2.2 (3d-Radar AS, Trondheim, Norway). The data were converted from frequency-domain to time-domain through an inverse fast Fourier transform by using a Kaiser window with a beta value of 6 [69] for a reduced bandwidth of 100–750 MHz, in order to eliminate low- and high-frequency noise. Because the data were recorded in the frequency-domain, the bandwidth can also be delimited during post-acquisition data processing based on the desired resolution and PD [26,70]. Background removal was done using a moving-mean filter over a window of 10–15 m along the scanlines in order to mitigate horizontal banding, especially targeting antenna-ringing noise [71]. For the conversion of the depth expressed in two-way travel time (ns) to depth expressed in distance (m), the time zero and RDP has to be estimated. The time zero is the time of the first arrival at the soil surface (including time for direct air and ground waves) and it was assumed to be constant over the entire area for each study site. The time zero was determined as the two-way travel time, where the first peak in signal magnitude was observed. The RDP was estimated from hyperbola fitting and evaluated by using values from the literature [71,72]. We refer to [70] for a more comprehensive overview of data processing for an SFCW GPR system.

**Figure 4:**
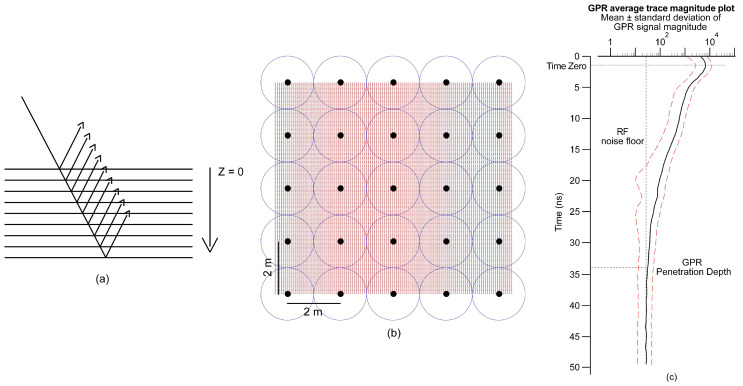
Illustration showing the procedure for calculating average global and localized 3D-GPR penetration depths: (**a**) equal reflectivity layered earth model, (**b**) 2 x 2 m^2^ regular grid points (black) and circles with 1 m radius (blue) overlain on densely sampled 3D-GPR data (red), and (**c**) average GPR trace magnitude plot as a function of time with background radio frequency noise floor and GPR penetration depth. The solid black line corresponds to the mean and the dashed red lines correspond to the mean ± standard deviation of the GPR signal magnitude. Note the logarithmic scale used to express the magnitude mean and standard deviation envelope.

As the Examiner software does not provide average trace magnitude (ATM) plots, the data were exported in ASCII format for further analysis of average global and localized PDs by using MATLAB software v R2019b (MathWorks Inc., Massachusetts, USA). The ATM plot is representative of the average GPR signal strength in function of time/depth and it was derived from calculating the mean and standard deviation (SD) of the signal magnitude at each two-way travel time step. It provides important information on coherent system noise, clipped GPR signal, choice of appropriate gain function, flat-lying reflectors, and, serving the main purpose here, on signal attenuation and PD [64]. Firstly, we assume an equal reflectivity layered earth model, i.e., an equal proportion of energy is reflected back to the surface from the RDP contrast in each infinitesimally thick layer boundaries encountered by the propagating GPR wave (Figure 4a). Instead of taking into account all measurement locations of the high-resolution 3D-GPR survey, the calculation of the ATM plots was limited to a subsample of the 3D data set in order to reduce the computation time. At each of the twelve study sites, subsampling was done based on the definition of a 2 × 2 m^2^ regular grid (Figure 4b). The average global PD over the entire study site was derived by both visual examination of the processed 3D-GPR data and from an ATM plot, including the single nearest-neighbor traces of each of the grid nodes. More specifically, when the ATM plot shows a stabilization of the decrease in mean magnitude, and associated SD, in two-way travel time/depth, it is assumed that the RF noise floor is reached and the corresponding time/depth is used as a measure for the PD of the 3D-GPR signal (Figure 4c). This is because, while the signal signature of the V-series air-coupled antenna arrays (3d-Radar AS, Trondheim, Norway) decays up to a certain time/depth and increases thereafter, making it possible to determine the PD by the computation of the automatic gain control [33,34], the signal signature of the DXG-series ground-coupled antenna arrays decays to a constant RF noise floor invalidating the use of the above approach in our study.

Moreover, the 3D-GPR system does not store the information of the recordings a few nanoseconds before the transmitter fires as some other time-domain GPR systems do, which can be useful for assessing the background RF noise floor. Hence, for the calculation of localized PD, an arbitrary threshold mean magnitude was assigned, depending on the expected background RF noise floor and the time at which five consecutive time samples were consistently below this threshold was assumed as the PD. The threshold value was chosen based on the mean magnitude recordings at the last few time steps. The localized PD was derived from calculating the ATM plot at each of the individual grid nodes, including all of the data points within 1 m radius (Figure 4b).

### 2.4. The DUALEM Instrument and Survey

The basic configuration of an EMI instrument consists of a transmitter (Tx) and receiver (Rx) coil pair. A primary magnetic field is generated by powering the Tx coil with an alternating current. This primary field generates eddy currents in the conductive material present in the subsurface generating a secondary magnetic field. The primary and secondary fields are both detected at the Rx coil. Because the primary field is known, the secondary field can be calculated relating the response to the actual subsurface properties [73]. The quadrature-phase and the in-phase signal response of an EMI sensor are representative of the EC_a_ and the magnetic susceptibility of the soil [74]. Each Tx-Rx coil array combination “sense” a different soil volume corresponding to different depth sensitivities that are dependent on the coil spacing (*S*) and array orientation [74,75,76,77]. Moreover, the depth sensitivities are non-linear [75,76,78]; hence, the measured EC_a_ is a depth-weighted response that complicates its interpretation in terms of the true EC variability [68,79]. The depth of exploration (DOE) for EC_a_ measurements can be arbitrarily defined as the depth at which the signal accumulates 70% of its total sensitivity and under low induction number (LIN) conditions, the DOE can be approximated to be a function of *S* and array orientation [74]. It should be noted that the LIN approximation is no longer valid in highly conductive (> 100 mS m^−1^) conditions and the actual depth of investigation (DOI) can vary significantly as a function of soil EC [74,76,79,80].

The DUALEM (Dualem Inc., Milton, ON, Canada) series are frequency-domain multi-receiver EMI sensors that operate on a single frequency of 9 kHz. The DUALEM-21S (Figure 3b) has two pairs of horizontal coplanar (HCP) and perpendicular (PRP) Tx-Rx coil configurations. The Tx is located at one end and is shared by all of the Rx coils, at a distance of 1.1 m and 2.1 m for the PRP coil configurations and 1 m and 2 m for the HCP configurations. The DOE of the PRP and HCP arrays are 0.5 *S* and 1.6 *S* respectively when the instrument is placed on the ground [81]. As such, the 1.1 m PRP and 1 m HCP configurations measure EC_a_ to depths of 0.5 and 1.6 m, respectively, while the 2.1 m PRP and 2 m HCP configurations provide EC_a_ down to depths of 1.0 and 3.2 m [78]. A DUALEM-421S sensor with an additional pair of receiver coils at 4.1 (4.1 m PRP) and 4 m (4 m HCP) spacing, which measure the EC_a_ down to depths of 2.0 m and 6.4 m, respectively, was used at the Fensholt lowland and Holtum sites. 

The sensors were mounted on a sled (0.3 m above the ground) behind an ATV and georeferencing was done by using an RTK/GNSS located in-between the 1-m Tx-Rx arrays for the DUALEM-21S and above the Tx coil in case of the DUALEM-421S. Sufficient distance (~ 3 m) was ensured between the instrument and the ATV to avoid interference with the measurement field. The sensor data were sampled at a rate of 2–10 Hz. Table 1 shows the dates and three-days total rainfall before the DUALEM surveys were performed at different sites. The DUALEM surveys were performed at a different time when compared to the 3D-GPR surveys and without consideration of ground wetness level from prior rainfall similar to the 3D-GPR surveys.

### 2.5. The DUALEM Data Processing and Inversion

The data processing and inversion were done by using the Aarhus Workbench software [82], which performs a fully nonlinear inversion routine with AarhusInv code [83]. The data processing involves both automatic and manual steps. In automatic data processing, the negative EC_a_ values were removed and the data from different channels were corrected for the RTK/GNSS offset by shifting their lateral position to the center of the Tx-Rx arrays along the driving line direction. The negative EC_a_ values occur either when the sensor’s quadrature-phase response falls below the noise level in a resistive terrain or due to the vicinity of strong lateral heterogeneities and needs to be removed before any interpretation, as they are indicative of equipment malfunction [73]. The data were then averaged by using a running mean width (3–6 m), depending on the in-situ soil variability to improve the SNR, and an appropriate sounding distance (1–3 m) was chosen based on the average sounding distance of the measured raw data to subsample the averaged data at uniform spacing. The soil variability was assessed based on the difference in EC_a_ measurements between consecutive soundings. A careful choice of running mean width and sounding distance are necessary in order not to smear the data generated by the soil variability at hand, yet to eliminate the redundant information to reduce the computation time for performing inversions. After the automatic processing, manual inspection of the raw data was done in order to identify and remove potential noise due to coupling with anthropogenic sources, such as buried cables, metal fences, or proximity of the instrument to the ATV when making turns. The changes made in the raw data were incorporated into the averaged data generated through the automatic processing step. We refer to [79] for a more comprehensive overview of the data processing of a DUALEM instrument using Aarhus Workbench software.

Later, an inversion of the processed EMI data was performed using a quasi-3D spatially constrained inversion algorithm, applying constraints both in-line and cross-line directions using Delaunay triangulation accounting for model consistency between proximal soundings [84]. The inversions were executed using a 10-layer model with depths to the top of each layer being 0, 0.2, 0.5, 0.9, 1.4, 2.2, 3.3, 4.8, 7.0, and 10.0 m, respectively, and with a uniform initial EC estimate of 25 mS m^−1^. A smooth type of model was chosen amongst other models (layered, blocky, sharp), as this requires the least stringent assumptions on the subsurface architecture. As a final step, quality control was accomplished by plotting the total residual, data residuals, and DOI in order to assess the quality of inversion. The inversion results, i.e. distributions of EC values over the entire depth interval considered, were used to calculate an average EC over 0–1.5 m depth in order to establish an easier comparison basis between the EMI and GPR results. This is the main depth interval of interest with respect to drainpipe location, as drain lines are usually installed at a depth of 1.0–1.5 m. Later, the EC (0–1.5 m) estimates were kriged using ordinary kriging [85] onto the same 2 x 2 m^2^ regular grid as used for the localized PDs for comparison.

## 3. Results

### 3.1. The 3D-GPR Results

**Figure 5:**
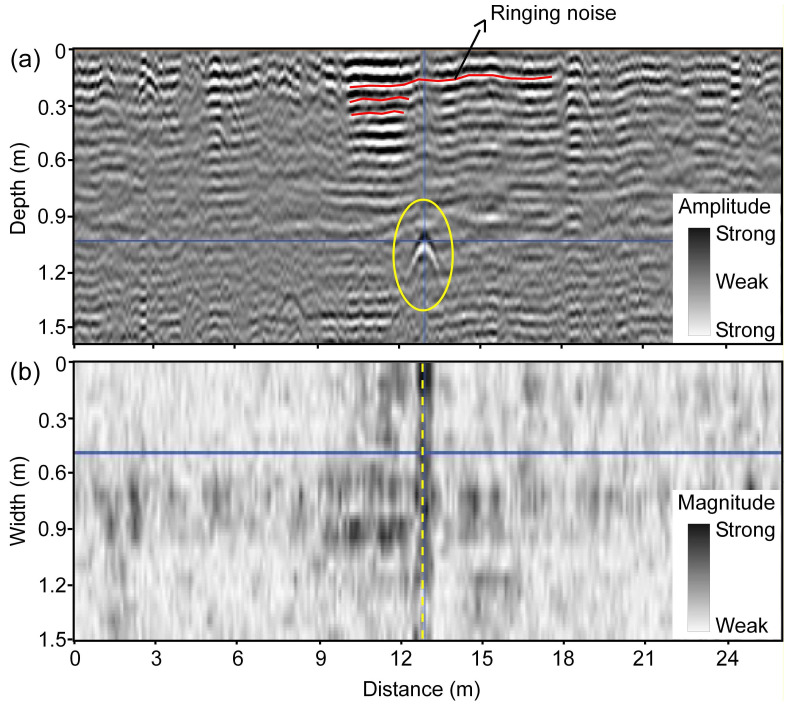
An example from Fensholt lowland showing the typical signature of a drain line marked in yellow when the 3D-GPR traverse is perpendicular to drain line orientation: (**a**) hyperbolic pattern in the vertical profile of reflections (amplitude) and (**b**) linear pattern in the horizontal slice (~1 m depth) of reflection strength (magnitude). The data were collected in January 2016.

**Figure 6:**
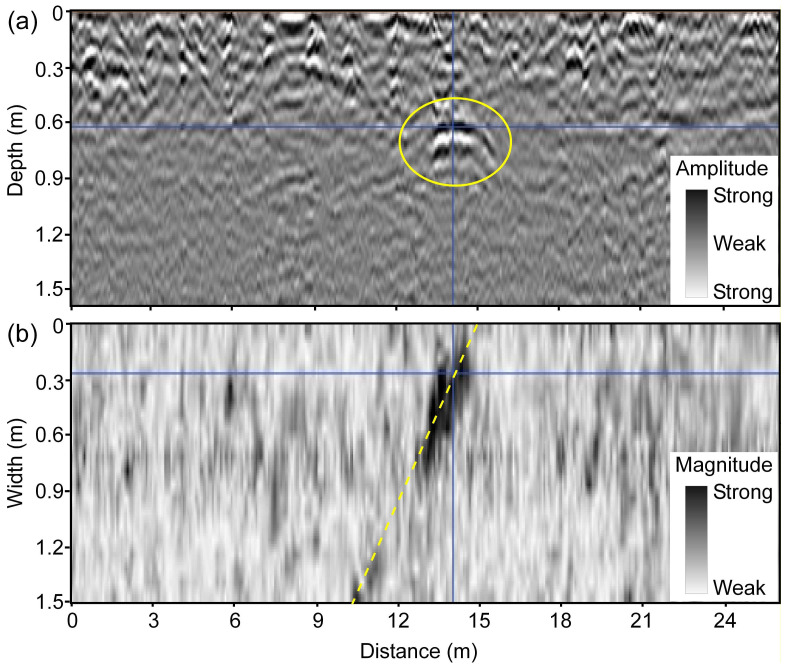
An example from Fensholt upland showing the typical signature of a drain line marked in yellow when the 3D-GPR traverse is at an angle to drain line orientation: (**a**) hard to recognize hyperbolic pattern in the vertical profile of reflections (amplitude) and (**b**) linear pattern in the horizontal slice (~0.6 m depth) of reflection strength (magnitude).

The typical signature of a drainage pipe in the 3D-GPR data is a hyperbolic pattern in vertical profiles and a linear pattern in horizontal time/depth slices (Figure 5) when the instrument is moved perpendicular to the drain line direction. This is because drainage pipes are cylindrical in shape and at their usual depth of installation (1.0–1.5 m) can be regarded as point size objects concerning to GPR survey along a perpendicular traverse. Thus, due to the fact that the GPR signal is propagated into the subsurface as an elongated cone of energy and “sees” buried features both in front of it and behind it [50,61,65], the arrival times of reflections retrace a hyperbolic pattern with the apex of the hyperbola coinciding with the location of the drain line and the extent of linear pattern in the horizontal slice representing the length of the drain line. As hyperbolic patterns can also be generated by other point size objects (rocks, cavities, etc.), the linear pattern of increased reflection strength in the horizontal slice indicates the presence of a two-dimensional (2D) linear object as expected for a drainage pipe and helps to eliminate false positives. When the GPR traverse is oblique to the drain line orientation, the hyperbolic pattern becomes horizontally spread out, depending on the angle of divergence from the perpendicular and, therefore, becomes more difficult to recognize in the vertical profiles (Figure 6). The drain line signature is that of a linear banded feature in the vertical profiles [15,17] when the GPR traverse is parallel to the drain line orientation. Here as well, in both cases, the presence of a 2D linear feature in the horizontal slices can sometimes be helpful to confirm the existence of a drainage pipe. This demonstrates the advantage of using the 3D-GPR with a wide antenna array swathe in comparison to the traditional GPR systems where data collection along discrete parallel and perpendicular transects according to a grid [15] or following spiral and serpentine patterns [17] is necessary in order to confirm the 2D nature of the anomaly and to ascertain its orientation.

**Figure 7:**
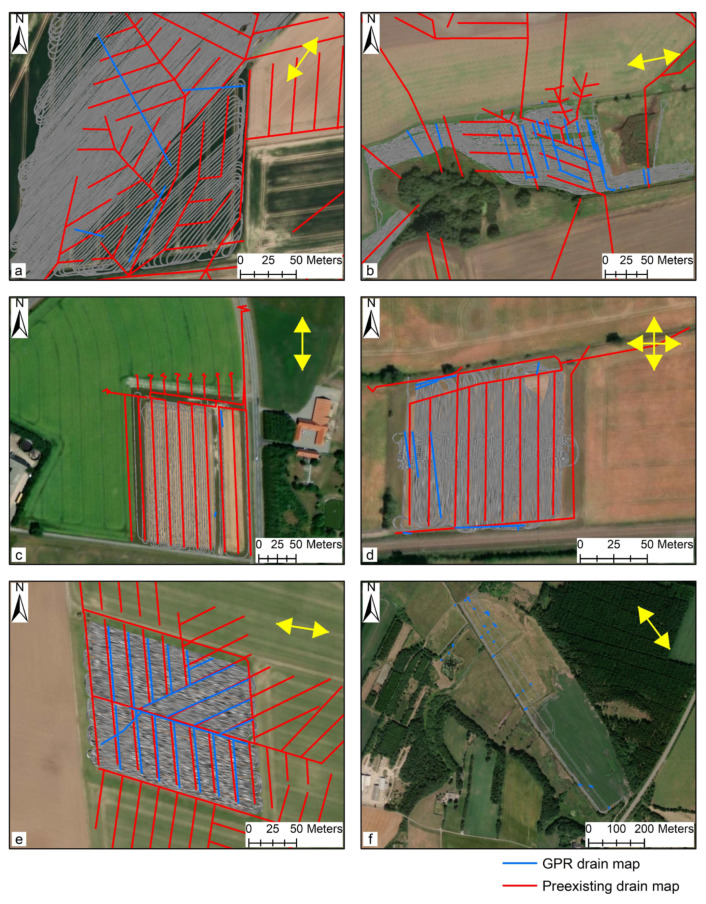

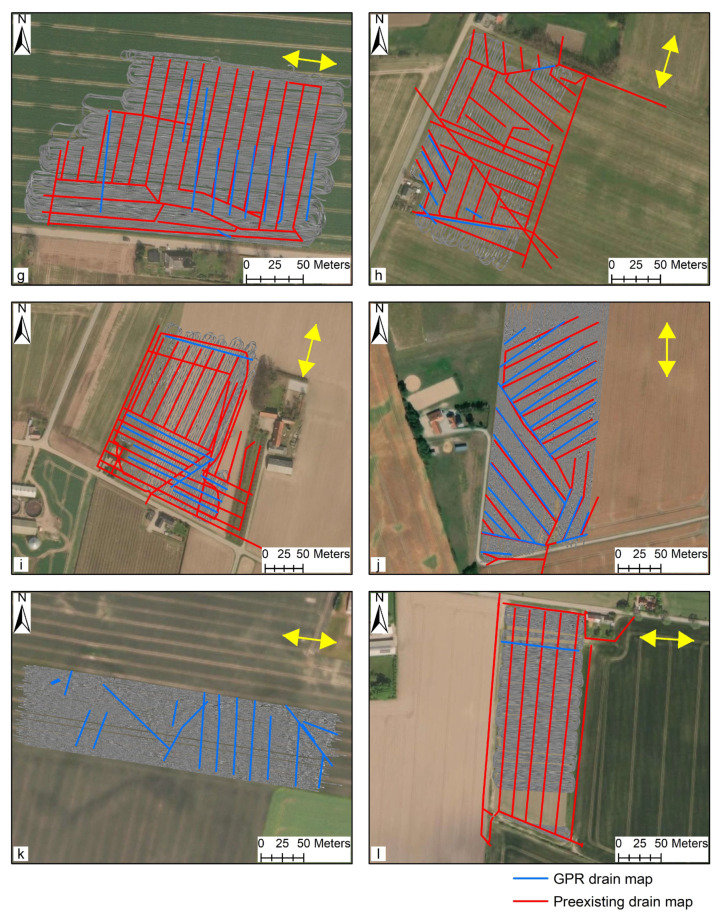
The drains mapped using the 3D-GPR instrument (blue) overlain on pre-existing drain maps (red) and the 3D-GPR survey transects at the different sites: (**a**) Fensholt upland, (**b**) Fensholt lowland, (**c**) Silstrup, (**d**) Estrup, (**e**) Faardrup, (**f**) Holtum, (**g**) Lillebæk-1, (**h**) Lillebæk-2, (**i**) Lillebæk-3, (**j**) Juelsgaard, (**k**) Kalundborg, and (**l**) Lund. The pre-existing drain map at the Holtum and Kalundborg sites were missing. Yellow arrows indicate the 3D-GPR survey directional trend and a base map provided by ArcMap 10.6 ([88]) was used as the background at all the sites.

**Figure 8:**
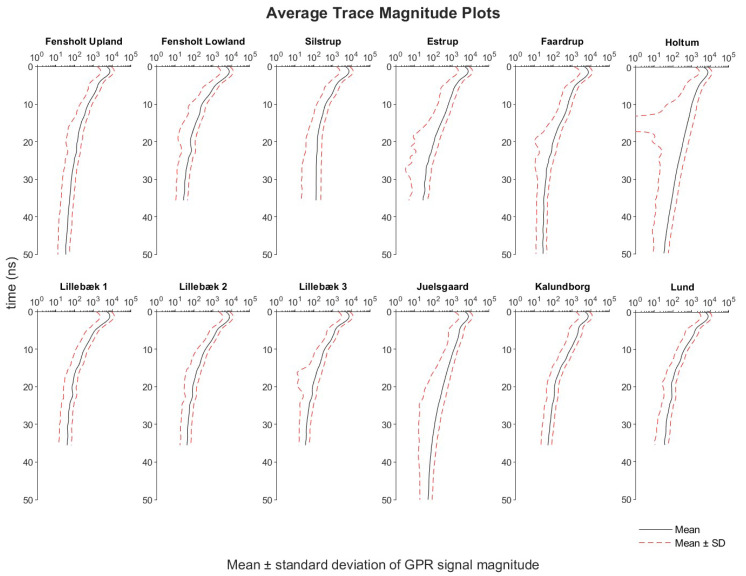
Average trace magnitude (ATM) plots for all the study sites.

Figure 7 shows drain lines mapped using the 3D-GPR overlain on the pre-existing drain maps provided by the farmers/landowners/site managers and the 3D-GPR survey transects at all of the study sites, except for Holtum and Kalundborg (Figure 7f,k). At Holtum, a pre-existing drain map was unavailable. At Kalundborg, it was not possible to georeference the drain card provided by the farmer due to the lack of control points that align with a referenced map, but an aerial photo captured by the Royal Air Force in 1954 (retrieved from [86]) provided an alternative reference background and shows the locations of the drain pipes (see later). The visibility of the drain pipe locations in this aerial image can be explained by its recording date soon after the drainage installations and/or after a sufficient rainfall event [87]. The drain card at Kalundborg is a typical example of how the drainage installations were documented back in the time (1950s). The drain cards mostly lack the georeferencing information, which causes the propagation of errors during the digitalization process. Additionally, the people (farmer/professional engineer) who plan the drainage design can be different from the drainage contractor who carries out the installations. This can lead to discrepancies between the pre-documented drain cards and the actual pattern followed due to practical difficulties incurred in the field during drainage installations. Some key observations, the expected drain line depths, and the estimated 3D-GPR global PD (Figure 8) at each study site are discussed below and summarized in Table 2.

Figure 7a,b show the drain lines mapped at Fensholt upland and lowland, respectively. At Fensholt upland, less success was observed in mapping the drain lines. While the hyperbolic patterns were not clearly visible in the vertical profiles (Figure 6), a few linear features of increased reflection strength were detected in the depth slices. The observed pipe-like patterns were at a shallow depth (0.4–0.8 m) and out of the four drains mapped in the upland area, only one closely aligns both in location and orientation to the pre-existing drain map. The lack of success can only partially be attributed to the limited penetration of the 3D-GPR signal here, as the farmer acquired the pre-existing map from different generations and was unsure whether the drainage installations were made as per the original plan. At Fensholt lowland, the mapped drains were at two different depths, which suggests that they probably date from two different installation periods. This can be attributed to the dynamic nature of the organic soils requiring periodical drainage reinstallations due to soil compaction and subsidence. Here, the shallow drains were located at 0.5–0.8 m depth and the deep drains at 1.0–1.5 m. The surveys performed on dry soil conditions, in August 2015 and September 2016, respectively, using a ground-coupled and an air-coupled antenna array, proved to be more suitable for drain line mapping as compared to the survey that was performed on wet soil with snow cover and a possible frozen topsoil layer in Jan. 2016. Although a better anomaly contrast and cleaner data were observed on the wet soil with snow cover, due to better ground coupling and a possible decrease in soil EC because of the partly frozen topsoil layer—both contributing to less signal attenuation, significant signal ringing was observed in the top few decimeters, which prevented the detection of the shallow drain lines. Figure 5 shows the ringing noise as horizontal banded features, yet these were generally largely removed by the background removal during data processing. The ringing noise can be explained by signal reverberations near the soil surface due to a high RDP contrast between the snow/frozen soil and wet soil underneath. The lower success rate in detecting the deep drains that corresponded with the pre-existing map is mainly due to a combination of a decrease in signal resolution with depth and a driving direction parallel to the drain line orientation. For the latter, the detectability of drainpipes is impeded, as it is harder to distinguish linear target features when the GPR is moved along the drain line orientation [15,17] and due to wave polarization [20,61]. Besides, there could sometimes be inherent differences between adjacent measurement channels, which can cause striping patterns limiting data interpretability. Two extra drainpipes that were located in the far west corner of the field were not shown in the figure. Detailed observations on the assessment of the suitability of the 3D-GPR for different survey configurations and site conditions along with the drains mapped in the Fensholt lowland area can be found in [68].

Figure 7c–e show the drains mapped at Silstrup, Estrup, and Faardrup. At Silstrup, none of the drains from the existing parallel drainage network were found. However, two small pipe-like patterns were observed outside the area of interest, close to the buffer zone [52]. The lack of success can be attributed to limited global PD (1.0–1.5) of the 3D-GPR signal at this site, as there were a few locations where the signal already started to vanish beyond 0.4–0.5 m depth. Moreover, the survey direction parallel to the drain lines orientation could also be problematic. This is both because of the problems aforementioned and the wide spacing between the adjacent transects, thereby having an incomplete coverage of the area and, hence, of the existing drainage network. At Estrup, only three pipe-like patterns were observed in the region of interest but they did not align well with the existing parallel drainage pattern. Nevertheless, the drains that were mapped in the buffer zone corresponded more closely with the pre-existing drain map. The drain along the southern boundary is a cut-off drain and it was installed to prevent outside water from entering the drainpipes in the test field; the drains along the northern boundary are part of a collector drain and a 10-cm diameter pipe draining the field to the west [52]. The poor success can again generally be attributed to the limited penetration of the 3D-GPR signal and the survey direction being parallel to the drain lines’ orientation in the data collected along the tillage direction. Here, the same as Fensholt lowland, as the data were collected along closely spaced transects there were very few locations where the data were missing at the locations of the expected drain lines. The data collected across the tillage direction and perpendicular to the drain line orientation resulted in more noise due to rough uneven surface conditions. At Faardrup, almost all of the pipes in a herringbone pattern were mapped; the collector drainpipe west of the area was only partially mapped. As before, the survey direction parallel to the drain line orientation hindered the drain detection. Furthermore, an extra pipe-like pattern was found in the southwest corner that was not marked on the pre-existing drain map. Only linear patterns of increased reflection strength were visible in the depth slices and the high success rate here can be attributed to the sufficient penetration of the 3D-GPR signal.

**Figure 9:**
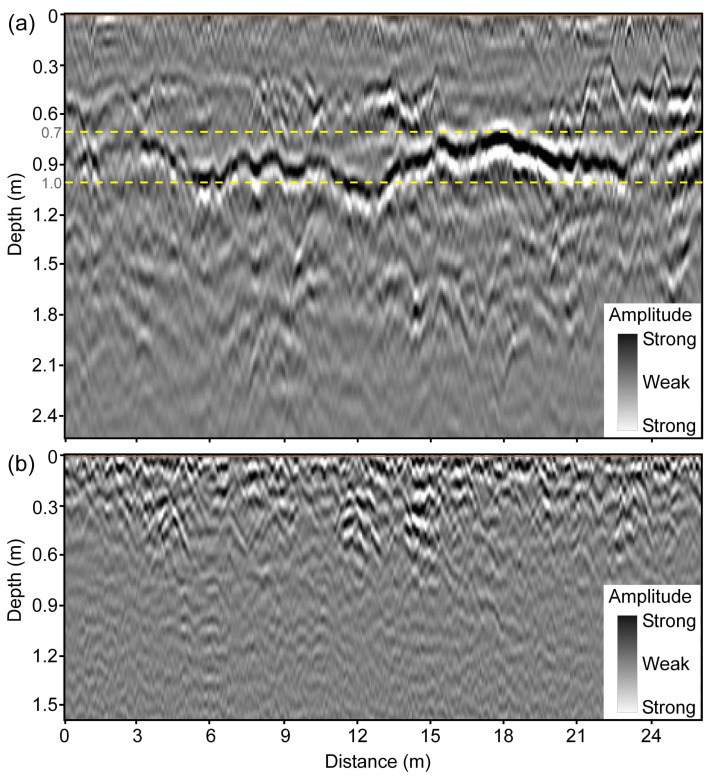
An example of the GPR profiles from the Holtum and Lillebæk-2 sites, respectively showing: (**a**) strong reflections from the soil layer boundary and deep penetration depth (~2.0 m) and (**b**) limited penetration depth (~0.6 m) of the 3D-GPR signal. The yellow lines were marked to indicate the depth of 0.7–1.0 m where a large SD was observed in the 3D-GPR signal magnitude at the Holtum site.

Figure 7f shows the drains that were mapped at Holtum. As only a limited area was surveyed, only 28 small drain segments were found spread across the larger area. Moreover, the lack of knowledge of the existing drainage network resulted in limited inferences about the success rate at this site. Nevertheless, the success rate is assumed to be high because of deep penetration of the 3D-GPR signal showing strong pedological reflectors down to 2 m depth. The Holtum site is one amongst the sites where we observed the deepest penetration (2.0–2.5 m) of the 3D-GPR signal (Table 2), hence a GPR profile is provided as an example in Figure 9a. In addition, a large SD was observed in the signal magnitude at a depth of 0.7–1.0 m (13–17 ns), as can be seen in the ATM plot (Figure 8), which can be explained by the occurrence of a soil layer boundary at this depth (Figure 9a).

Figure 7g–i show the drains mapped at Lillebæk sites 1, 2, and 3, respectively. The pre-existing maps show that the drainage pattern at these sites is complex. At Lillebæk-1, only the drain lines in approximately north-south orientation were located, except for a small pipe-like segment to the southern part of the field. This was because the survey direction was approximately in an east-west orientation. This was also the case at Lillebæk-2 and 3. At Lillebæk-2, we were only partly successful in mapping the drain lines either perpendicular or at an angle to the survey direction, especially to the southern part of the field, while comparatively more success was achieved at Lillebæk-3. At both Lillebæk-2 and 3, the same as Silstrup, a wider survey transect spacing was used and we partially missed collecting data at the expected drain line locations partly explaining why we failed to map the entire network of drain lines oriented parallel to the survey direction. Overall, limited penetration of the 3D-GPR signal was observed at all three sites as the signal mostly starts to disappear near 0.5–0.6 m depth, except for a few locations where reflectors down to 1.2 m were observed, hence we were less successful in mapping the drain lines. Lillebæk sites are amongst the sites where we observed the shallowest penetration (0.6–1.2 m) of the 3D-GPR signal (Table 2), hence a GPR profile is provided as an example in Figure 9b. Moreover, only subtle anomalies of drain line signatures were observed in the depth slices and they were hard to recognize in the vertical profiles.

**Figure 10:**
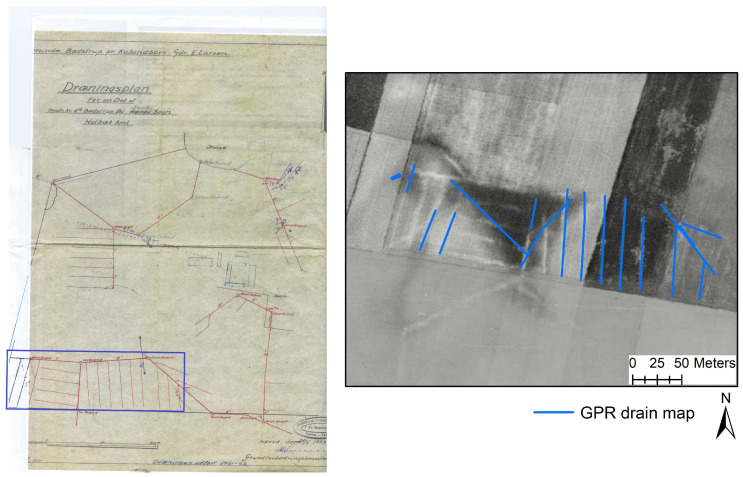
Example from Kalundborg site showing: (**a**) drain card received from the farmer with the 3D-GPR survey region marked in blue (courtesy: Rasmus Erik Eriksen) and (**b**) drains mapped using the 3D-GPR overlain on the aerial imagery captured by the Royal Air Force in 1954.

Figure 7j–l show the drain lines mapped at Juelsgaard, Kalundborg, and Lund, respectively. High success was achieved at Juelsgaard and Kalundborg, while poor to no success was observed at Lund. At Juelsgaard, most of the drain lines in the complex pattern were mapped except for the drains with a north-south orientation—the survey having been carried out in north-south orientation as well. The collector drains were only partly mapped, both those in the northwest-southeast and northeast-southwest orientations. At Kalundborg, the position and orientation of the drains mapped with the 3D-GPR matches with the drains visible in the historical aerial photo (background) in the eastern part of the field (Figure 10b). In the western part, the 3D-GPR was less successful in finding the drain lines and the pipe-like patterns identified did not correspond well with the drain card or with the aerial photo (Figure 10). While the drains orienting in an approximately north-south orientation were only partly mapped, a strange pipe-like pattern trending northwest-southeast orientation was noticed in the location where the expected drainage orientation was east-west trending. This might be a drain pipe extending from the neighboring drainage network, as can be seen in the background aerial imagery but was absent in the drain card (Figure 10). Again, the lack of success (in the western part of the field) was because of the driving direction being parallel to the drain lines orientation. In addition, limited inferences can be made regarding the PD at Kalundborg, as the data were collected using a shorter time window (36 ns, 1.5 m). At Lund, only one pipe-like pattern was observed and that too parallel to the driving direction. This drain line signature does not align with the existing parallel network and the lack of success can be attributed to the limited penetration of the 3D-GPR signal.

All in all, a high success rate was achieved in mapping the drain pipes at five sites—Fenholt lowland, Faardrup, Holtum, Juelgaard, and Kalundborg, whereas the other seven sites were less successful. The deviations in location and/or orientation were observed between the drains mapped by the 3D-GPR and the pre-existing drain maps, which can be addressed to uncertainties associated with the different processes from drainage design to documentation of the installations, and digitalization afterwards. The overall detection success rate generally relates to the survey direction relative to the drain lines orientation and PD of the 3D-GPR signal. In this respect, for the latter, the soil EC measured at these sites could provide us with a better understanding of the electromagnetic signal attenuation.

### 3.2. The DUALEM Results

In agricultural applications, the EMI measurements are mainly influenced by soil physio-chemical properties, such as soluble salts, clay content and mineralogy, soil water content, bulk density, organic matter, and soil temperature; consequently, making EC_a_ measurements a suitable proxy to map spatial variation of several edaphic properties [43,44,45,46]. The correlation between EC_a_ measurements and different soil properties is often site-specific and for non-saline soils, the soil texture and soil wetness can be regarded as the predominant factors that influence the measured EC_a_ [42,43]. Moreover, as the soil texture is a static factor, consistent spatial patterns can be observed over time, and, hence, the below interpretations were made based on the assumption that the effects of soil moisture were subordinate to soil texture.

Table 3 shows the mean EC_a_ obtained for all the considered coil configurations and mean EC (0–1.5 m) obtained after the inversion of the EMI measurements. The organic soil of Fensholt lowland has notably higher mean EC_a_ and EC values as compared to the mineral soil of the upland area. At Silstrup and Faardrup, the mean EC_a_ was low in the 1.1 m PRP and significantly increased for the 2.1 m PRP, 1 m HCP, and 2 m HCP signals. This is because, at these sites, the sandy loam topsoil is underlain by clay till and sandy clay till, respectively. Similar mean EC values were observed at both of the sites. A similar interpretation can be made for the Estrup site, although the mean EC_a_ and EC were generally high when compared to the other sites. The sites at Lillebæk-1, 2, and 3 have similar mean EC_a_ and EC values relating to similar soil properties at all these sites. The mean EC_a_ and EC values were very low at Holtum (4.9–9.0 mS m^−1^), because the soil was predominantly sandy. Relatively low mean EC_a_ and EC values were also observed at Juelsgaard and Kalundborg. At Juelsgaard, this was because of loamy sand to sandy loam material, with an intermediate layer of coarse sand underlain by clay till subsoil. At Kalundborg, the values were slightly higher due to the intermediate layer of organic material. At Lund, similar mean EC_a_ and EC values were observed as in for the Fensholt upland, which can be attributed to the clayey sand topsoil and clay till in the subsoil. In general, the EC_a_ values gradually increase with the DOE of the channels, which implies that at all of the sites the soil EC generally increases with depth. In addition, the mean EC estimates showed higher values when compared to the shallow measuring signals (1.1 m PRP, 2.1 m PRP, and 1 m HCP). This was coherent to the observation that was made by [79], who noted that the inversions expose the true dynamic range of the conductivity distribution and, hence, the average inverse modeled EC can be larger than the EC_a_ values directly recovered from the different measurement configurations. As mentioned earlier, this is because the depth sensitivities are non-linear which complicates the direct interpretation of the EC_a_ measurements in terms of the vertical distribution of EC values. Overall, low mean EC (< 15 mS m^−1^) values were observed at Holtum, Jueslgaard, and Kalundborg, and moderate mean EC (20–30 mS m^−1^) values were noticed at Fensholt upland, Silstrup, Faardrup, Lillebæk-1, 2, and 3, and Lund. At Fensholt lowland and Estrup, the mean EC (> 30 mS m^−1^) values were relatively high when compared to all other sites. These observations were consistent with the above-described differences in soil type.

## 4. Discussion

### 4.1. Combined Interpretation and Localized 3D-GPR Penetration Depth

A combined interpretation was made to understand the influence of soil EC on GPR signal attenuation and, hence, the estimated PD. Deeper signal penetration (~ 2 m) was observed at Holtum with a low mean EC (0–1.5 m) value. Correspondingly, a high success rate was achieved in finding the drain lines. The same reasoning applies to the Kalundborg and Juelsgaard sites. A moderate mean EC was observed at Faardrup; nevertheless, a high success rate was achieved due to sufficient penetration of the 3D-GPR signal. Poor to no success was observed in finding the drain lines at the other sites with moderate mean EC values due to the limited penetration of the 3D-GPR signal. Evidently, at Estrup with a relatively high mean EC, limited penetration of the 3D-GPR signal resulted in a low success rate. Contrastingly, a high success rate was observed at Fensholt lowland even with a high value of mean EC. This can be explained by the shallow depth of the drainpipes at Fensholt lowland in comparison to the other sites causing the smaller PD of the 3D-GPR signal to be still sufficient. 

**Figure 11:**
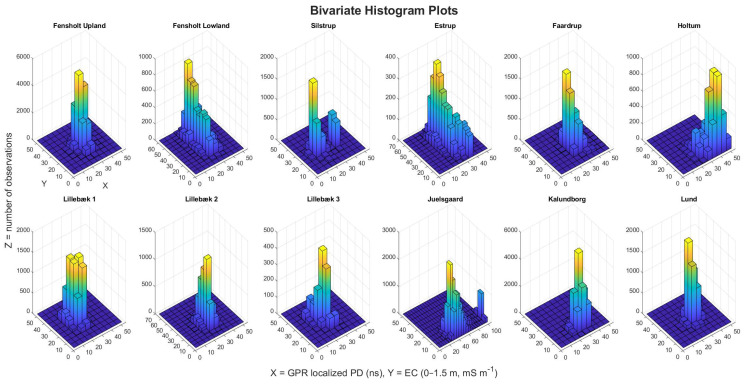
Bivariate histogram plots showing the comparison between the 3D-GPR localized penetration depth (ns) and EC (0–1.5 m, mS m^−1^).

We calculated the 3D-GPR localized PDs at all the study sites in order to investigate the theoretically expected effect of EC on GPR signal attenuation and PD. Table 4 and Figure 11 gives the summary statistics, Spearman’s ρ, and bivariate histogram plots of the localized PD (in ns) and kriged EC values for all of the sites. The localized PD and EC were both binned with a bin width of five for their entire range of distribution and plotted together for comparison. At Fensholt upland, the SD was less in both localized PD and EC, resulting in a concentrated density of observations in the bivariate histogram plots thereby suggesting that the soil is fairly uniform across the site. At Fensholt lowland, the mean localized PD was similar to Fensholt upland, even though the EC values were in the higher range (11–60 mS m^−1^). This can be attributed to good coupling of antenna array with the ground and lack of strong pedological reflectors, except for a sandy ridge structure [68], thereby energy loss is mostly only due to signal attenuation that is caused by soil EC. At Silstrup, a bi-modal distribution was observed in the localized PDs. The two peaks in the density of observations were at a similar range of EC values. Hence, this can be associated with the other factors that influence the GPR SNR such as the difference in antenna array coupling with the ground while acquiring data across different regions and may not relate to the soil physical properties. At Estrup, higher PDs were observed at low EC values and lower PDs were observed at high EC values. This corresponds well with the notion that soil EC controls the GPR PD. At Faardrup, again relatively less variation (low SD) was observed in both the localized PD and EC values. At Holtum, an overall deeper penetration of the GPR signal was observed, which can be attributed to the low (mean of 10.2 mS m^−1^) EC values observed at this site. Here, the variability in localized PD at similar EC values can be explained from the loss of energy due to reflections from the pedological layer boundary. At Lillebæk-1, 2, and 3, the distribution of observations was in a similar range both for localized PD and EC, supported by the similar soil type at these sites. Again, at Juelsgaard and Kalundborg, the correspondence between deeper penetration of the GPR signals and low EC is confirmed. While a bimodal distribution was observed at Juelsgaard with the highest SD and localized PD was estimated to be ~100 ns at a few locations, limited inferences can be made about the localized PD at Kalundborg as the 3D-GPR data was collected over a shorter time window. At Lund, the distribution of both the localized PD and EC was more localized similar to Fensholt upland, and Faardrup and a low SD was again observed. Overall, at all of the sites, except for Fensholt upland, the distribution of the localized PDs was observed in a similar range as the average global PDs and the maximum of the localized PD matches the time window of the processed 3D-GPR data. At Fensholt upland, the mean localized PD was on a slightly higher range when compared to the average global PD and the maximum is smaller than the time window. These can be caveats caused by assigning arbitrary thresholds for mean signal magnitude at RF noise floor as the derivations of PD are, in general, quite tricky and inherently subjective, inducing bias. Hence, caution should be exercised in order to not over-interpret the data.

Overall, very weak to no correlations (ρ) were observed between the localized PD and mean EC at Fensholt upland and lowland, Lillebæk-1, 2, Juelsgaard, Kalundborg, and Lund sites. Weak correlations were observed at Silstrup and Faardrup, while moderate correlations were observed at Estrup, Holtum, and Lillebæk-3 sites. The correlations were significant at all of the sites, except for Fensholt upland and Kalundborg (P-value < 0.05, Table 4). The interpretation of correlation strength was based on [89] and, as expected, an inverse relationship was observed at the sites where the observed correlation was prominent. Moreover, when considering that positive auto-correlations generally exist for spatial datasets, the classification of correlation strengths made here should be interpreted with caution for the correlation assessment [85].

**Figure 12:**
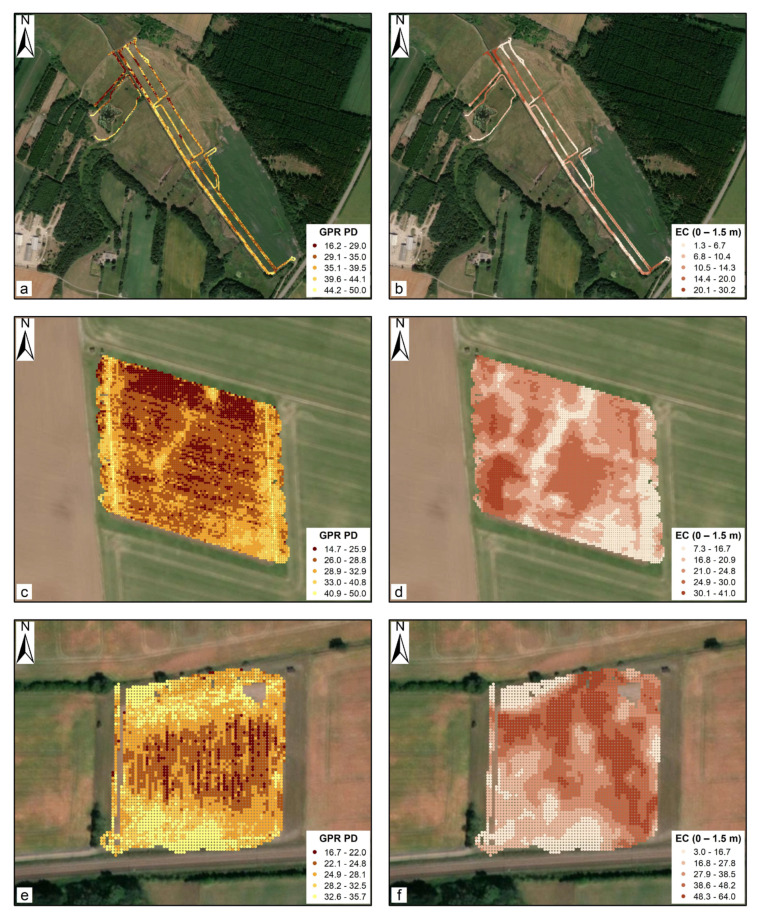
Maps showing the comparison between the 3D-GPR localized penetration depth (ns) and EC (0–1.5 m, mS m^−1^) at Holtum (**a**,**b**), Faardrup (**c**,**d**), and Estrup (**e**,**f**). The color bar definitions rely on a classification of the data according to Jenks natural breaks optimization in ArcMap 10.6 ([88]).

To further exemplify, Figure 12 shows one study site from each of the three categories, i.e. low mean EC (Holtum), moderate mean EC (Faardrup), and high mean EC (Estrup), where visible correlations can be observed between the 3D-GPR localized PD and the EC. At Holtum, good correspondence was observed between the 3D-GPR localized PD and mean EC values: a larger PD coincides with a low mean EC and vice versa across most of the area. At Faardrup, a good correspondence was found in the central and southeastern parts of the site with low mean EC values. At the rest of the locations, this was only partially true, as no clear correspondence was observed between the localized PD and EC. Moreover, consistently low PDs were observed in the northern part of the site and they belong to one regional survey recording. This can be attributed to the poor coupling of antenna array with the ground while recording the dataset. At Estrup, the expected relation was overall confirmed, especially to the northwest and southwest parts of the site where low mean EC values were observed. Yet, some deviation appears in the northern part of the site. The striping patterns in the survey direction in localized PD calculations at Faardrup and Estrup (Figure 12c,e) could be because of the channels recording asynchronously or due to the difference in surface conditions when driving in line with the till direction causing differences in antenna array coupling with the ground. At the rest of the sites except for Lillebæk-3, the expected correspondence between localized PD and mean EC was visually less straightforward to confirm, demanding a more sophisticated derivation of the localized PD. In addition, it should be kept in mind that the comparisons were made with one possible EC model representative of the true EC distribution and the uncertainties that are associated with the inversion routine need to be taken into account.

### 4.2. Recommendations and Future Work

We obtained maximum success in finding the drain lines in the 3D-GPR surveys that were carried out either perpendicular or at an angle to the expected drain line orientation. This was both because it was harder to distinguish linear banded features in the vertical profiles when driving parallel to the drain lines’ orientation and because of the non-spatially comprehensive coverage of the sites and, hence, of the expected drain line locations. Therefore, any a priori available information on the expected drainage pattern and orientation can be a good lead to plan the GPR survey for drain line mapping when visiting a new site. At the sites where the pre-existing drain maps are available, it is recommendable to survey in the direction perpendicular to the expected drain line orientation, as it is more likely to traverse over the top of the drain lines thereby increasing the chances of detecting them; with an easier-to-recognize drainpipe signature (i.e. hyperbolic pattern in the vertical profiles). Additionally, this direction is to be generally preferred because of wave polarization [20,61]. However, for site situations where this is not a possibility because of the surface conditions, it is recommendable to at least ensure to obtain full spatial coverage of the region of interest, in order not to miss data collection at the expected drain line locations. At the sites, where a priori information is unavailable, it is advisable to carry out a few preliminary transects in mutually orthogonal directions to get a sense of the drain lines direction akin to [15,17] before performing the actual full-scale survey. In this way, the preliminary survey alleviates the problem of not detecting any drain lines and it can give an understanding of the expected GPR penetration and drain line depths at the site, which are the two main factors that determine whether GPR would work for drain line detection.

Moreover, a high success rate in drain line detection was observed in low EC predominantly sandy soils as compared to high EC clay-rich soils. Therefore, any information on the soil type or availability of EC maps for the site can provide an idea of expected GPR signal attenuation. This can be key information to predetermine if GPR would be a suitable technology to map the drain lines at a particular site. In general, wet soil could have a substantially higher EC, thereby reducing the GPR signal penetration. Therefore, it is recommendable to carry out the GPR surveys preferably on dry soil conditions. On the other hand, in soils where EC does not vary significantly with water content, there will be a large RDP contrast between wet soil and air-filled pipes, which would result in a greater amount of radar energy being reflected off the pipe and returning to the receiving antennas [15,16]. Hence, if possible, it is advisable to plan the EMI and GPR surveys concurrently or at least during the same season for comparison, as soil wetness conditions can have a significant impact on their measurements. This was a caveat in our study, as, in some cases, the EMI and GPR surveys are years apart and not within the same season, and they were performed without consideration of the optimal environmental conditions. Besides, as the hyperbolic patterns were hard to recognize at some of the sites, it would be advantageous to assess the soil water content with the use of a time domain reflectometer for the accurate conversion of the GPR two-way travel time to depth expressed in the distance.

Efforts moving forward, the future research aims at predicting the suitability of the GPR for subsurface drainage mapping by using electromagnetic simulation software, such as gprMax [90] based on EMI measurements for different GPR antenna configurations, varying site conditions, and drain line depths. The computer modelling can be useful to pre-emptively decide whether GPR technology is appropriate for drain line mapping at a particular site, provided the other information is known. Besides, additional methods (drone imagery, magnetic gradiometer) will be tested and their complementary usage will be analyzed, for example [87,91], with the goal of providing guidelines in relation to the choice of sensor.

## 5. Conclusions

The mapping of agricultural subsurface drainage systems is important for agronomic, economic, and environmental reasons. In this study, we tested the use of an SFCW 3D-GPR with a wide antenna array for subsurface drainage mapping and evaluated its performance by using an EMI instrument. A high success rate was achieved in finding the drain lines at five sites using the 3-D GPR, but the results at the other seven sites were less successful. We were particularly successful in mapping the drain lines oriented either perpendicular or at an angle to the 3D-GPR survey direction. The discrepancies and offset between the actual location of the drains that were mapped by the 3D-GPR and the pre-existing drain maps can be related to the inaccuracies associated with the processes involved from drainage design to documentation of the installations and the digitalization later on. The inverse relationship between both the average global and localized PDs of the 3D-GPR versus mean EC (0–1.5 m) was generally confirmed, corroborating the expected influence of EC on signal attenuation. Hence, the EC measured by the EMI sensor can act as ancillary information to explain the success that is achieved by the GPR in finding the drain lines, complementarily providing information on spatial variability of soil properties that are of importance to precision agriculture. The main novelty of this work lies in showcasing the use of a 3D-GPR for subsurface drainage mapping and the corresponding derivation of the 3D-GPR global and localized PDs.

## Figures and Tables

**Table 1:** Summary of the study sites’ location, soil type, dates of the ground penetrating radar (GPR) and electromagnetic induction (EMI) surveys, and total precipitation (in brackets) three-days prior to the surveys.

Study Site	Location Coordinates *		Soil Type	Date of the GPR Surveys and 3-Days Prior Rainfall ^#^ (mm)	Date of the EMI Surveys and 3-Days Prior Rainfall ^#^ (mm)
	Northing (m)	Easting (m)			
Fensholt upland	6205900	568885	Sandy/silty clay loam overlain on clay till	20 September 2016 (0)	3 September 2014 (0.3)
Fensholt lowland	6204718	567145	Organic soil overlain on clay till	18 August 2015 (16.4); 21 January 2016 (0.7); 21 September 2016 (0)	10 September 2015 (0)
Silstrup	6309890	478431	Sandy clay loam/sandy loam topsoil overlain on clay till	12 November 2015 (19.2)	16 May 2011 (5.0)
Estrup	6148875	504378	Sandy loam topsoil overlain on clay till	12 November 2015 (14.0); 28 September 2017 (0.8); 14 August 2018 (34.6)	5 September 2011 (0.2)
Faardrup	6132550	648662	Loam/sandy loam topsoil overlain on sandy clay till	9 September 2015 (9.2)	28 July 2011 (3.1)
Holtum	6204566	520304	Sand and silt	22 January 2016 (3.1)	8 October 2015 (13.6)
Lillebæk-1	6109780	610730	Sand-mixed clay	24 August 2015 (0)	1 September 2015 (14.3)
Lillebæk-2	6110380	611557	Sand-mixed clay	24 August 2015 (0)	1 September 2015 (14.3)
Lillebæk-3	6109685	611347	Sand-mixed clay	24 August 2015 (0)	1 September 2015 (14.3)
Juelsgaard	6256750	533900	Loamy sand topsoil overlain on coarse sand, sandy loam and clay till	21 November 2018 (1.0)	22 September 2017 (8.3)
Kalundborg	6168000	632470	Sandy loam topsoil overlain on intermediate layer of organic material and sandy clay till	15 August 2018 (43.0)	18 August 2016 (0)
Lund	6127000	709200	Clayey sand topsoil overlain on clay till	28 August 2017 (4.4)	14 September 2016 (0)

* All the coordinates are in UTM (wgs84, zone 32N). ^#^ Rainfall data obtained from the Danish Meteorological Institute weather archive [59].

**Table 2:** Summary of the time zero, estimated relative dielectric permittivity (RDP), success rate, estimated drainage depth, and average 3D-GPR global penetration depth (PD) at different sites.

Study Site	Time Zero (ns)	Estimated RDP	Success Rate (%)	Estimated Drainage Depth		3D-GPR Global PD	
				(ns)	(m)	(ns)	(m)
Fensholt upland	1.2	12	10	10–18	0.4–0.8	13–24	0.5–1.0
Fensholt lowland *	1.3	10	75	12–18 22–33	0.5–0.8 1.0–1.5	22–33	1.0–1.5
Silstrup	1.3	10	0	15–22	0.7–1.0	22–33	1.0–1.5
Estrup *	1.3	12	5	17–29	0.7–1.2	24–36	1.0–1.5
Faardrup	1.5	10	99	14–20	0.6–0.9	23–35	1.0–1.5
Holtum	1.5	6	High ^#^	10–39	0.5–2.3	34–42	2.0–2.5
Lillebæk-1	1.3	10	25	9–16	0.4–0.7	14–27	0.6–1.2
Lillebæk-2	1.5	10	15	10–17	0.4–0.7	14–27	0.6–1.2
Lillebæk-3	1.3	10	25	9–16	0.4–0.7	14–27	0.6–1.2
Juelsgaard	1.3	12	90	20–29	0.8–1.2	48–59	2.0–2.5
Kalundborg	1.3	12	70	10–25	0.4–1.0	24–36	1.0–1.5
Lund	1.2	12	0	15	0.6	15–29	0.6–1.2

* Time zero, estimated RDP, and 3D-GPR penetration depth were calculated for the data collected in Aug 2015 for Fensholt lowland and Nov 2015 for Estrup. ^#^ Presumed to be high due to lack of pre-existing drain maps.

**Table 3:** Mean EC_a_ measured by 1 m PRP (0–0.5 m), 1 m HCP (0–1.6 m), 2 m PRP (0–1 m), 2 m HCP (0–6.4 m), 4 m PRP (0–2 m), and 4 m HCP (0–6.4 m) coil configurations after removing the negative values and mean EC (0–1.5 m) estimates after data processing and inversion.

Study Site	1 m PRP	1 m HCP	2 m PRP	2 m HCP	4 m PRP	4 m HCP	EC (0–1.5 m)
	(mS m^−1^)						
Fensholt upland	10.4	17.7	16.5	23.7	X	X	22.3
Fensholt lowland *	14.2	22.3	20.6	26.7	23.8	22.2	32.2
Silstrup	7.6	18.2	15.3	22.7	X	X	22.7
Estrup	12.9	28.6	23.3	35.2	X	X	33.0
Faardrup	7.7	14.8	14.3	19.0	X	X	21.3
Holtum *	4.9	5.9	6.0	8.3	9.0	6.9	9.0
Lillebæk-1	12.1	21.1	19.2	27.5	X	X	26.4
Lillebæk-2	10.6	20.0	18.1	27.4	X	X	24.8
Lillebæk-3	10.4	20.8	18.4	29.0	X	X	24.9
Juelsgaard	4.6	6.7	7.6	12.3	X	X	9.3
Kalundborg	6.0	11.3	10.4	17.6	X	X	13.2
Lund	10.0	16.0	16.1	21.2	X	X	23.0

* DUALEM-421S was used at these sites; DUALEM-21S was used at the other sites. X No data available for the respective coil configuration.

**Table 4:** Summary statistics and Spearman’s rank correlation coefficient (ρ) of the 3D-GPR localized PD and mean EC (0–1.5 m) calculated onto a 2 × 2 m^2^ regular grid at all the study sites.

Study Site	3D-GPR Localized PD (ns)				EC (0–1.5 m, mS m^−1^)				ρ	*p*-Value
	Min	Max	Mean	SD	Min	Max	Mean	SD		
Fensholt upland	11	49	27.6	3.3	9	38	25.0	4.2	−0.00	0.9516
Fensholt lowland	15	36	27.8	4.3	11	60	38.0	11.9	0.09	<0.0001 *
Silstrup	8	36	21.4	8.6	7	32	23.0	3.0	−0.20	<0.0001 *
Estrup	17	36	27.5	4.3	3	64	33.9	14.5	−0.41	<0.0001 *
Faardrup	15	50	28.7	3.8	7	41	21.8	4.8	−0.38	<0.0001 *
Holtum	16	50	36.7	6.4	1	30	10.2	4.9	−0.50	<0.0001 *
Lillebæk-1	6	36	21.7	3.0	14	41	26.6	5.2	−0.19	<0.0001 *
Lillebæk-2	15	36	26.3	3.2	11	65	24.8	6.4	−0.11	<0.0001 *
Lillebæk-3	10	36	23.4	3.9	11	40	25.1	5.8	−0.53	<0.0001 *
Juelsgaard	16	100	48.1	17.6	3	20	9.2	2.7	−0.05	<0.0001 *
Kalundborg	8	36	29.5	3.7	6	25	13.5	2.6	−0.00	0.9559
Lund	14	36	27.3	2.9	15	32	23.1	3.0	0.03	0.0101 *

Min—Minimum; Max—Maximum; SD—Standard deviation; *p*—Probability. * Indicates statistically significant correlations.
